# Influence of Land Use and Point Source Pollution on Water Quality in a Developed Region: A Case Study in Shunde, China

**DOI:** 10.3390/ijerph15010051

**Published:** 2017-12-30

**Authors:** Wenjing Bo, Xiaoke Wang, Qianqian Zhang, Yi Xiao, Zhiyun Ouyang

**Affiliations:** 1 State Key Laboratory for Urban and Regional Ecology, Research Center for Eco-Environmental Sciences, Chinese Academy of Sciences, Haidian District, Beijing 100085, China; bowenjing9871@163.com (W.B.); xiaoyi@rcees.ac.cn (Y.X.); zyouyang@rcees.ac.cn (Z.O.); 2 University of Chinese Academy of Sciences, Beijing 100049, China; 3 Institute of Hydrogeology and Environmental Geology, Chinese Academy of Geological Sciences, Shijiazhuang 050061, China; z_qqian@163.com

**Keywords:** water quality, land use, point source of pollution, Shunde

## Abstract

To design and implement policy to manage water quality, it is important to investigate land use and possible sources of pollution. In this study, using Pearson regression analysis, redundancy analysis and multiple regression analysis, we assess the influence of land use and point sources on water quality in the river system in Shunde district in 2000 and 2010. The results show that water quality was related positively with water surface but negatively with impervious and urban greening area. Additionally, water quality was related negatively to point source emissions of chemical oxygen demand (COD) and ammonium-nitrogen (NH_4_-N). The total explanatory power of spatial variation of water quality was improved from 43.4% to 60.0% in 2000 and from 31.3% to 57.8% in 2010, respectively, when the influence of point sources was added into redundancy analysis between water quality and land use. Thus, both land use management and point source pollution control should be considered for improving river water quality.

## 1. Introduction

River water quality is strongly influenced by land use [1,2]. Many studies have reported that most water pollutants such as particles, nutrients and metals show a significantly positive correlation with the percentage of construction land and a significantly negative correlation with percent woodland [3,4,5,6]. Cropland cover, however, may have a more complex relationship with water quality. The increase of farmland coverage was found to increase the concentrations of both nitrate and sulfate ion in some case studies [7], but to not influence others [8]. Other emerging types of land use, such as nursery garden and urban green land, have received relatively little attention in research on water quality and their effects could be underestimated. Previous studies have shown that land use close to a river was a better predictor of water quality than the spatial pattern of the entire watershed [9,10]. In addition, in many regions with flat relief and canals, it is impossible to clearly delineate the watershed boundary. Therefore, in this study, we created a wide range of buffer zones to analyze the effect of land use adjacent to rivers on water quality. However, the relationships between land use and water quality vary significantly, because watershed characteristics and point source pollution vary across different regions [11,12]. Study areas with point source pollution might weaken the relationships between land use and water quality [5,11]. In this study, we integrate point source pollution emission into correlation analysis of land use and water quality, aiming to assess how point source pollution affects river water quality.

Shunde district is a very developed region in the Pearl River delta, the Gross domestic product (GDP) reached CNY 279.3 billion in 2016 which ranks first amongst 987 municipal districts in China. It has shifted from a traditional agriculture-based economy to a manufactured-based economy [13]. With industrialization and urbanization, a significant amount of cultivated land has been converted to built-up land. From 2000 to 2010, cropland decreased from 139.5 to 12.7 km^2^, whilst impervious surface increased from 300.2 to 371.3 km^2^. Urbanization and population growth lead to increased point and non-point source pollutant emission in Shunde district [14]. There were 12,000 manufacturing enterprises such as appliance manufacture, metal manufacturing and furniture manufacturing. A large amount of sewage was discharged into rivers, which increased from 17.68 million metric tons in 2000 to 93.49 million metric tons in 2010 [15]. Both this land use change and point source of pollution may impact water quality.

The objectives of this work are to delineate the changes of water quality in Shunde district between 2000 and 2010 and reveal the roles of non-point source pollution, changing land use pattern and point source pollution on water quality.

## 2. Materials and Methods

### 2.1. Study Area

This research covers Shunde district (22°40′ N–23°2′ N, 113°1′ E–113°23′ E), located in the central part of the Pearl River Delta, Guangdong Province, Southern China. Shunde district has a subtropical monsoon climate, with long summers and short winters. The average annual temperature and precipitation are 21.9 °C and 1639 mm, respectively. Shunde district has 16 main rivers and over 120 streams flow through this area and make up a 215 km^2^ network of dikes and ponds, which constitute 26.7% of the total area of Shunde.

### 2.2. Data Sources

**Figure 1:**
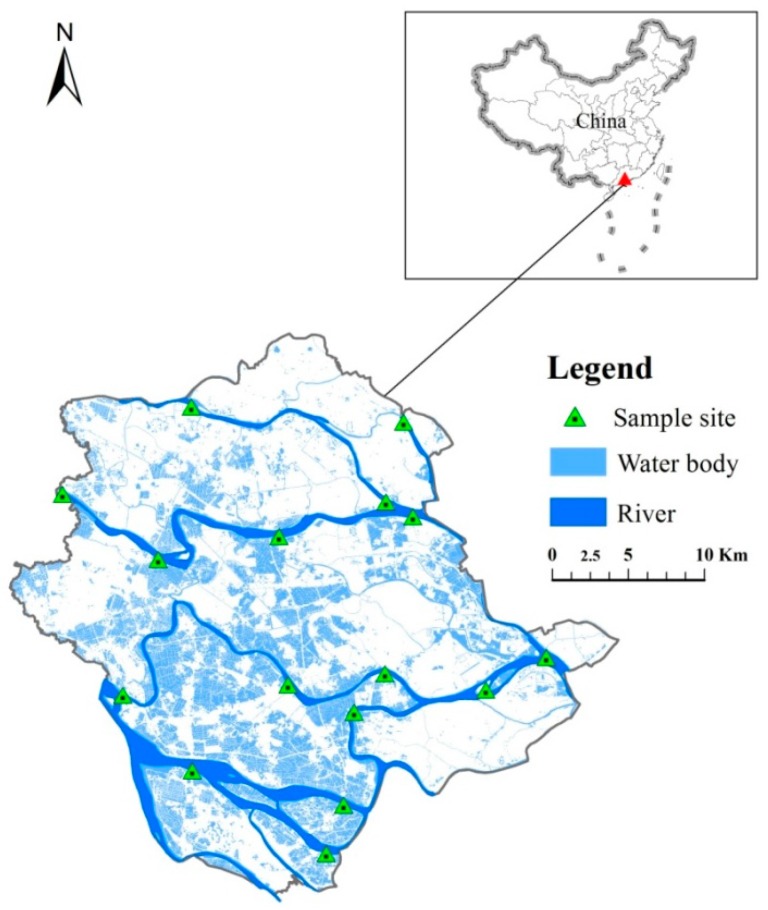
The spatial distribution of the 16 sample sites in study area of Shunde district.

Water quality at 16 sites (Figure 1)were monitored monthly in 2000 and 2010 by Shunde Environmental Monitoring Center, according to protocols of surface water quality sampling and analysis issued by the Chinese State Environment Protection Agency [16]. The 16 sites are located in the five main waterways in Shunde district, namely Ronggui, Shunde branch waterway, Tanzhou, Donghai and the Shunde waterway. Six water quality parameters were monitored: pH, dissolved oxygen (DO), permanganate index (COD_Mn_), biochemical oxygen demand (BOD), ammonium-nitrogen (NH_4_-N) and fluoride. Besides these parameters, five heavy metals contents have also been monitored but not assessed in this study because their concentrations are below the limits of their detective values.

Land use data with resolutions of 30 m in 2000 and 2010 were produced from satellite Landsat 5 Thematic Mapper images and Moderate Resolution Imaging Spectro-radiometer by the Institute of Remote Sensing Applications, Chinese Academy of Sciences. Land use was classified into nine types: forest, shrub, pond, river, cropland, nursery garden, impervious surface, urban green land and bare land.

**Figure A1:**
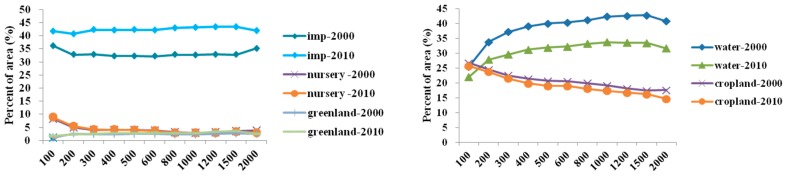
Mean value of land use characteristics of the river section within the buffer of 100 m–2000 m in 2000 and 2010.

To quantify the effects of land use on river water quality, eleven buffer zones (100, 200, 300, 400, 500, 600, 800, 1000, 1200, 1500, and 2000 m) within the range of 1000 m upstream and 100 m downstream were created using Arcgis 10.2 [4]. To generate reproducible data, the 2000 m buffer zone was chosen as land use is easier to predict in this zone (Appendix Figure A1).

The location (latitude and longitude) discharge amounts, COD values and NH_4_-N emissions from point sources in 2000 and 2010 were provided by Shunde Environmental Monitoring Center. The annual emissions of COD and NH_4_-N increased from 0.35 to 0.011 billion tons to 0.58 and 0.018 billion tons, respectively, between 2000 and 2010. The point sources within a 2000 m buffer zone were used to quantify the effects of point source pollution on river water quality.

### 2.3. Statistical Analysis

Many statistical tests have been widely applied to determine the relationship between land use and water quality, such as correlation analysis [3,4,7,17,18], multiple regression [5,19], principal component analysis [5,20] and redundancy analysis [2,4]. In this study, the changes in water quality between 2000 and 2010 were assessed by t-test. Pearson correlation analysis and redundancy analysis were performed to test the quantitative relationship between land use within a 2000 m buffer zone and water quality parameters. In the analysis, the dependent parameter is water quality, and the independent parameter is type of land use. Multiple regression analysis was used to test the relationships between variables, point source pollutant emission, land use type and water quality in 2000 and 2010. Before using redundancy analysis, detrended correspondence analysis was employed to determine which method was more suitable, a linear (redundancy analysis) or a unimodal ordination method, canonical correspondence analysis as recommended by [21]. As the detrended correspondence analysis gradient shaft length was less than 3, redundancy analysis was used to determine the relationship between land use types, point sources emission and water quality in 2000 and 2010. Pearson correlation analysis, t-test and multiple regression analysis were carried out with SPSS 19.0 (IBM, Armonk NY, USA) for Windows. Detrended correspondence analysis and redundancy analysis were performed with CANOCO 4.5 (Microcomputer Power, Ithaca, NY, USA) for Windows.

## 3. Results

### 3.1. Water Quality Changed between 2000 and 2010

Water quality parameters changed markedly from 2000 to 2010 (Table 1). pH value significantly (*p* < 0.01) decreased from 7.83 in 2000 to 7.52 in 2010. COD_Mn_ significantly (*p* < 0.05) decreased from 2.35 mg/L in 2000 to 2.19 mg/L in 2010, while BOD, NH_4_-N and fluoride significantly (*p* < 0.01) increased from 1.31, 0.20 and 0.18 mg/L in 2000, respectively, to 2.05, 0.39 and 0.31 mg/L, respectively, in 2010.

### 3.2. Impact of Land Use Characteristics on Water Quality

**Figure 2:**
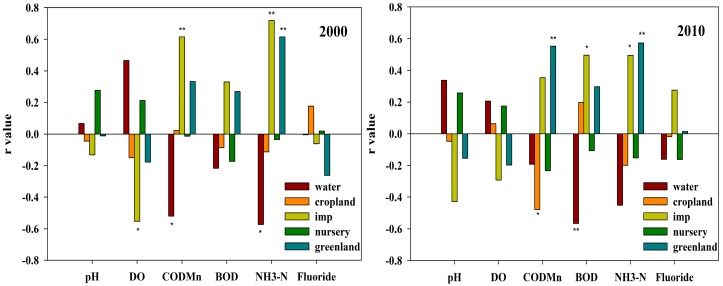
Pearson relationships between water quality and land use types within the 2000 m buffer in 2000 and 2010. “*” and “**” refer that the coefficients (r) are significant at the 0.05 level and at the 0.01 level, respectively.

Pearson regression analysis suggested the proportion of water, impervious land, green land and cropland are major factors influencing water quality (Figure 2). In 2000, the impervious surface and green land were negatively correlated with water quality. The proportion of impervious surface was correlated negatively with DO and positively with COD_Mn_ and NH_4_-N; the proportion of green land was correlated positively with NH_4_-N. However, the proportion of water surface was correlated negatively with COD_Mn_ and NH_4_-N.

In 2010, the proportion of impervious surface, green land, water surface and cropland affect the water quality (Figure 2). Pearson regression analysis showed that the proportion of impervious surface was positively correlated with BOD and NH_4_-N and the proportion of green land was correlated positively with COD_Mn_. However, the water surface and cropland improved water quality. The proportion of water surface was negatively correlated with BOD and the proportion of cropland was negatively correlated with COD_Mn_.

### 3.3. Impacts of Land Use and Point Source Pollution on Water Quality

Redundancy analysis was used to assess the impacts of land use and point source pollution within a 2000 m buffer on water quality in 2000 and 2010. Total explanatory power significantly increased from 31.3% to 57.8% when COD and NH_4_-N emissions were included in redundancy analysis in 2010 and from 43.4% to 60.0% when COD emission were included in redundancy analysis in 2000 (Table 2), indicating that point sources pollution have important impacts on water quality.

**Figure 3:**
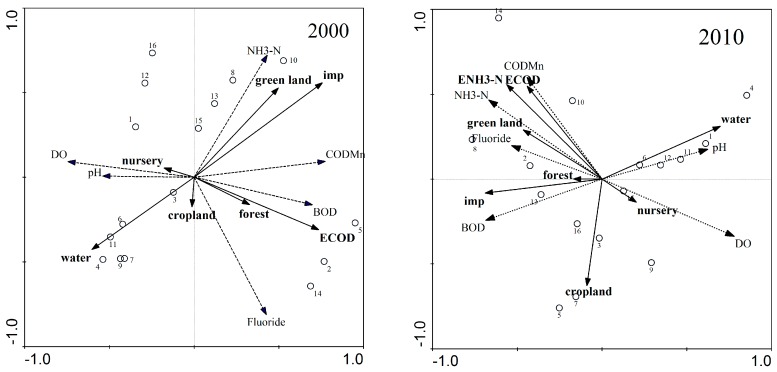
Redundancy analysis results of land use and water quality at 2000 m buffer in 2000 and 2010. “imp” refers to impervious surface; “DO”, “COD_Mn_”, “BOD” and “NH_4_-N” refer to dissolved oxygen; permanganate index; biochemical oxygen demand and ammonium-nitrogen, respectively. “E_COD_” and “E_NH4-N_” refer to chemical oxygen demand emission and ammonium-nitrogen emission.

In Figure 3, redundancy analysis results of land use and water quality are presented. COD_Mn_ and NH_4_-N concentrations were closely related to the proportions of urban green and impervious land and BOD was closely related with COD emission and proportion of forest in 2000. COD_Mn_ and NH_4_-N were closed related with COD and NH_4_-N emissions and urban green and impervious land in 2010.

Multiple regression analysis also indicated that COD_Mn_ and NH_4_-N emissions were important factors influencing water quality because they were positively related with COD_Mn_ (*p* < 0.05), NH_4_-N (*p* < 0.01) but negatively related with DO (*p* < 0.01) in 2010. A similar case was observed in 2000: COD_Mn_ emission was an important factor on water quality because it was positively related to COD_Mn_ (*p* < 0.05), BOD (*p* < 0.05), fluoride (*p* < 0.05) and negatively related to DO (*p* < 0.05).

Land use within a buffer zone also influenced water quality (Table 3). In 2000, the proportion of impervious surface was very positively correlated with COD_Mn_ (*p* < 0.05), NH_4_-N (*p* < 0.05). In 2010, the proportion of urban green land was significantly positively correlated with COD_Mn_ (*p* < 0.05).

## 4. Discussion

### 4.1. Effects of Land Use on River Water Quality

Urbanization in rapidly developing areas has led to an increase in impervious surfaces including roads, roofs, parking lots, sidewalks and a decrease in surfaces that can absorb and purify rainstorm runoff [22]. In our results, changes in the proportion of impervious surfaces showed a positive relationship with BOD, NH_4_-N and COD_Mn_ and a negative relationship with DO. This is consistent with previous studies which indicate that an impervious surface plays an important role in reducing water quality in adjacent aquatic systems [7,8,20,23,24]. An impervious surface can change natural hydrological conditions by increasing the volume and the velocity of storm runoff [25] and preventing natural percolation [22]. Storm runoff in urban areas contains various pollutants from residential and industrial areas [26,27,28], flows into rivers and reduces water quality [29]. Previous investigations have showed that the COD_Mn_, BOD and NH_4_-N content of storm water runoff from impervious surfaces were higher than other land use types in Shunde district [30,31].

The proportion of cropland was negatively correlated with COD_Mn_, which is consistent with the literature [8,32]. The major crop in Shunde district is green forage, which accounted for 54% of total cultivated area in 2010 [33]. Cropland composed of green forage can intercept some pollutants in storm runoff that is destined for the waterways. Therefore, we believe that planting green forage can intercept some pollutants near the river in Shunde district.

Previous research has shown that surface water negatively correlates with water quality [4]. Hydrological and biogeochemical processes in the water surface near the river are physically and biochemically connected to the water-quality in downstream rivers [34]. Our analysis, however, indicated that surface water negatively correlated with COD_Mn_, NH_4_-N and BOD, which may be due to the dilution and biodegradation of the pollutants [35]. Therefore, we argue that wetland including swamps, lakes and ponds can purify pollutants in the river basin in Shunde district.

It was determined that urban green land was positively correlated with NH_4_-N and COD_Mn_, which is consistent with results of other studies [36]. The intensive management of urban green areas, such as watering, fertilizing and spraying insecticide have negative effects on water quality [37]. Therefore, anthropogenic management of urban green land influences water quality. Hence, it is important to choose plant species that require less management and use lower levels of pesticides and fertilizers in the riparian zone.

Prolonged exposure to fluoride at high levels can lead to health problem [38]. In our study, the land use types within riparian zone have little effect on Fluoride (Figure 2). Many previous studies have reported that fluoride was released mainly from industrial sources, such as electrolytic extraction of aluminum ore, smelting iron ore, ceramics production and so on [39,40]. Our results also showed that the fluoride content of river in Shunde district is closely related to point source pollutant emissions in 2000 and 2010 (Table 3).

### 4.2. Influence of Point Source Pollution on Water Quality

Numerous studies have indicated that the relationships between land use and water quality do exist although it may vary with space and time because of different watershed characteristics and point sources of pollution [18,41]. Point sources of pollution might weaken the relationships between land use and water quality [5,11]. In our results, the total explanatory power significantly increased when point source pollution was added to redundancy analysis, indicating point source of pollution has an important impact on water quality. Our study also showed that COD and NH_4_-N emissions were positively correlated with COD_Mn_, NH_4_-N and Fluoride but negatively correlated with DO (Table 3). Therefore, point source control is important for improving river water quality in the developed region.

## 5. Conclusions

This study highlights that there is a correlation between adjacent land use types and water quality in Shunde river district. The Pearson correlation analysis indicated that the impervious surface was positively correlated with BOD, COD_Mn_, NH_4_-N and negatively with DO. However, water surface was negatively correlated with BOD and NH_4_-N. The water quality of rivers in Shunde district declined between 2000 and 2010 mainly due to changes in land use and emission of point source pollutants. The percentage of water surface significantly decreased and the proportion of impervious surface significantly increased from 2000 to 2010. Redundancy analysis indicated that point source pollutant emissions as well as adjacent land use types had important impacts on water quality. Multiple regression analysis indicated that an impervious surface correlated positively with COD_Mn_, NH_4_-N and negatively with DO. In addition to point source pollutants, COD and NH_4_-N emissions were positive correlated with COD_Mn_, BOD, NH_4_-N and fluoride. Although many factors may influence water quality in rivers, in this study, we demonstrated how point source pollution influences water quality and highlights a relationship between land use and water quality in a rapidly developing district of China.

## Figures and Tables

**Table 1:** Mean value and *t*-test results of water quality variables at the sample sites of Shunde district between 2000 and 2010.

Variables	2000	2010	2000–2010	
			*t*-Statistic	Significance
pH	7.83 ± 0.05	7.52 ± 0.12	8.71	<0.01
DO	6.82 ± 0.21	6.84 ± 0.34	−0.15	0.87
COD_Mn_	2.35 ± 0.13	2.19 ± 0.13	2.16	0.04
BOD	1.31 ± 0.24	2.05 ± 0.20	−5.56	<0.01
NH_4_-N	0.20 ± 0.09	0.39 ± 0.12	−5.12	<0.01
Fluoride	0.18 ± 0.08	0.31 ± 0.02	−6.70	<0.01

DO: dissolved oxygen; COD_Mn_: permanganate index; BOD: biochemical oxygen demand; NH_4_-N: ammonium-nitrogen.

**Table 2:** The dominant land use groups with the maximum explanatory power within 2000 m buffer scale in 2000 and 2010.

Year	Dominant Variable	Land Use			Dominant Variable	Point Source Pollution + Land Use		
		Cumulative Explained Variance (%)	Total Explained Variance (%)	*p*-Value		Cumulative Explained Variance (%)	Total Explained Variance (%)	*p*-Value
2000	Imp	29.3	43.4	0.01	imp	29.3	60.0	0.01
	Forest	31.9			E_COD_	44.9		
	Nursery	38.4			forest	49.0		
	Cropland	41.3			green land	54.3		
2010	Imp	14.4	31.3	0.09	water	22.6	57.8	0.03
	Green land	19.7			E_NH4-N_	42.3		
	Cropland	23.9			green land	45.9		
	Nursery	25.0			E_COD_	48.5		

Imp: impervious surface; E_COD_: chemical oxygen demand emission; E_NH4-N_: ammonium-nitrogen emission.

**Table 3:** Multiple regression analysis between land use types in 2000 m buffer, point source pollution emissions and water quality indicators.

Indicator	First		Second		Third		R^2^ Adj
	Factor	Std. Coef.	Factor	Std. Coef.	Factor	Std. Coef.	
COD_Mn__2000	Imp	0.47 *	E_COD_	0.43 *	--	--	0.472
COD_Mn__2010	Green land	0.51 *	E_COD_	0.48 *	--	--	0.472
BOD_2000	E_COD_	0.59 **	Green land	0.31	Forest	0.22	0.331
BOD_2010	Nursery	−1.58	Forest	1.41	Green land	1.05	0.274
NH_4_-N_2000	Imp	0.91 **	Forest	−0.48	Nursery	0.41	0.536
NH_4_-N_2010	E_NH4-N_	1.94 *	E_COD_	−1.39	Green land	1.11	0.568
DO_2000	Forest	−0.48	E_COD_	−0.42	Nursery	0.39	0.439
DO_2010	E_NH4-N_	−0.81 **	Cropland	−0.23	Green land	−0.19	0.537
Fluoride_2000	E_COD_	0.62 *	Imp	−0.57	Water	−0.38	0.323
Fluoride_2010	E_NH4-N_	1.56	E_COD_	−0.98	-	-	0.338

* Coefficient is significant at the 0.05 level; ** Coefficient is significant at the 0.01 level. “Std. Coef.” is the abbreviation of standardized coefficients. “imp” refers to impervious surface; “DO”, “COD_Mn_”, “BOD” and “NH_4_-N” refer to dissolved oxygen; permanganate index; biochemical oxygen demand and ammonium-nitrogen, respectively. “E_COD_” and “E_NH4-N_” refer to chemical oxygen demand emission and ammonium-nitrogen emission, respectively.
